# Supplementary material for: Test case sampling optimization for safety validation of automated driving systems
Source: Nat Commun. 2026 Feb 24;17:3114. doi: 10.1038/s41467-026-69675-8 (PMC13039333; doi:10.1038/s41467-026-69675-8)
Supplement: Supplementary file 1 — Supplementary Information [file 41467_2026_69675_MOESM1_ESM.pdf]

Supplementary Information
Test Case Sampling Optimization for Safety Validation of Automated Driving Systems

Chen Qian[1,†], Jingbin Xu[2,†], Xin Xing[3], Feng Guo[3,4,*]

[1]Dalian University of Technology, School of Economics and Management, Dalian, China; [2]Dalian University of Technology, School of Mechanical Engineering, Dalian, China; [3]Virginia Tech, Department of Statistics, Blacksburg, VA, USA; [4]Virginia Tech Transportation Institute, Blacksburg, VA, USA; *Corresponding author: feng.guo@vt.edu; †Equally contributing

Numerical Simulations

This section implements a Monte Carlo simulation on different data generation processes to evaluate the performance of our proposed method in comparison with other state-of-the-art approaches. We utilized the Gaussian kernel in our proposed KTCS algorithm, with hyperparameters set as suggested in[1]. The competitive methods are as follows: the Uniform subsampling (Uniform), Support Point (SP)[2], SPARTAN[3], the MMD-Critic[4], MED[5]. Simulations were conducted in Python (version 3.12.7). We performed 200 independent Monte Carlo replications.

To introduce non-linearity, we use a latent generative model, we first generate a synthetic dataset $\boldsymbol{x}_1, \ldots, \boldsymbol{x}_N$ in $\mathbb{R}^d$ by drawing lower-dimensional latent vectors $\boldsymbol{x}_1^*, \ldots, \boldsymbol{x}_N^*$ from a simple distribution (e.g., a Gaussian mixture), where each $\boldsymbol{x}_i^* \in \mathbb{R}^{d_*}$ with $d_* < d$. We then apply a nonlinear transformation to these latents via $\boldsymbol{x}_i^\top = A(\boldsymbol{W}\boldsymbol{x}{^*}_i^\top)$ where $\boldsymbol{W} \in \mathbb{R}^{d \times d_*}$ is a linear projection and $A(\cdot)$ is a nonlinear activation function. This process generates a dataset with a highly complex, nonlinear structure. For instance,[6] demonstrate that a similar generation scheme can produce richly detailed images.

In practice, safety-critical events are not only rare but also tend to lie far from the center and exhibit great diversity[7]. To mimic this pattern, we partition the latent generation into two components: a *major* component with many cases and low variance, and a *minor* component with few cases and high variance. The centers of *major* and *minor* latents are positioned far apart to reflect their distinct distributions.

For each synthetic dataset, we apply our proposed method and five competitive methods to select $0.5\sqrt{N}, \sqrt{N}, 2\sqrt{N}$ points. We evaluate performance using Information Potential (IP) and Maximum Mean Discrepancy (MMD). We also compute the proportion of minor modes covered (PCT) in the subsequent analysis. Intuitively, higher PCT values indicate better coverage of corner cases. For example, if there are 10 minor modes and a method selects at least one point from 8 of these modes, the value of PCT is 8.

Table 1. Performance comparison under simulation example 1.

			IP			MMD			PCT		
N	d	Method	$0.5\sqrt{N}$	$\sqrt{N}$	$2\sqrt{N}$	$0.5\sqrt{N}$	$\sqrt{N}$	$2\sqrt{N}$	$0.5\sqrt{N}$	$\sqrt{N}$	$2\sqrt{N}$
1100	20	**KTCS**	0.184	0.153	0.129	0.038	0.011	0.004	0.566	0.722	0.905
		MED	0.121	0.120	0.120	0.036	0.019	0.014	0.987	1.000	1.000
		MMD-Critic	0.143	0.135	0.146	0.026	0.012	0.014	0.727	0.997	1.000
		SP	0.169	0.148	0.137	0.011	0.006	0.004	0.107	0.271	0.520
		SPARTAN	0.221	0.188	0.168	0.052	0.026	0.012	0.134	0.251	0.442
		Uniform	0.223	0.187	0.168	0.054	0.025	0.012	0.137	0.261	0.467
1100	50	**KTCS**	0.130	0.100	0.085	0.056	0.023	0.008	0.688	0.899	0.982
		MED	0.079	0.070	0.073	0.051	0.025	0.016	0.998	1.000	1.000
		MMD-Critic	0.096	0.084	0.089	0.039	0.019	0.014	0.723	0.999	1.000
		SP	0.120	0.103	0.095	0.012	0.005	0.003	0.080	0.223	0.444
		SPARTAN	0.165	0.136	0.121	0.055	0.029	0.014	0.123	0.238	0.400
		Uniform	0.163	0.132	0.118	0.055	0.026	0.013	0.146	0.269	0.479
5500	20	**KTCS**	0.146	0.129	0.118	0.010	0.002	0.000	0.837	0.949	0.984
		MED	0.113	0.113	0.111	0.024	0.020	0.017	1.000	1.000	1.000
		MMD-Critic	0.130	0.146	0.172	0.013	0.018	0.037	0.998	0.998	1.000
		SP	0.151	0.145	0.138	0.004	0.002	0.001	0.265	0.482	0.760
		SPARTAN	0.184	0.166	0.154	0.022	0.011	0.006	0.270	0.493	0.748
		Uniform	0.184	0.166	0.154	0.023	0.011	0.006	0.284	0.498	0.741
5500	50	**KTCS**	0.103	0.087	0.080	0.016	0.004	0.000	0.850	0.988	0.995
		MED	0.065	0.067	0.070	0.031	0.024	0.020	1.000	1.000	1.000
		MMD-Critic	0.079	0.088	0.114	0.021	0.016	0.029	1.000	1.000	1.000
		SP	0.104	0.101	0.098	0.004	0.002	0.001	0.244	0.449	0.767
		SPARTAN	0.129	0.118	0.110	0.023	0.012	0.006	0.293	0.479	0.734
		Uniform	0.131	0.117	0.110	0.024	0.012	0.006	0.282	0.488	0.736

Example 1*:* We generate latents from a two-dimensional ring-shaped Gaussian mixture distribution. Specifically, for each component $k \in$ [Major, Minor], we draw $\boldsymbol{x}^*_{i,k} \sim \sum_{j=1}^{10} 0.1 \times \mathcal{N}\left(\mu_{j,k}, \Sigma_k\right)$, so that both the Major and Minor parts each have 10 modes. The mode centers are $\boldsymbol{\mu}_{j,\text{Major}} = \left(2\cos(\frac{j\pi}{10}), 2\sin(\frac{j\pi}{10})\right)$ and $\boldsymbol{\mu}_{j,\text{Minor}} = \left(4\cos(\frac{j\pi}{10}), 4\sin(\frac{j\pi}{10})\right)$ for $j = 1, ..., 10$. We set the covariance matrices as

$$\Sigma_{\text{Major}} = \begin{pmatrix} 0.02 & 0.02\rho \\ 0.02\rho & 0.02 \end{pmatrix} \quad \text{and} \quad \Sigma_{\text{Minor}} = \begin{pmatrix} 0.1 & 0.1\rho \\ 0.1\rho & 0.1 \end{pmatrix} \tag{1}$$

where ρ controls the feature correlation.

We set the ratio of Major to Minor sample at 10:1 and consider sample sizes of $N = 5500$ and $N = 1100$. For $N = 5500$, 5000 points originate from the Major component (500 points per mode) and 500 from the Minor component (50 points per mode). In our simulations, we use $\rho = 0.5$ and dimension $d = 20$ and 50. The entries for the transformation matrix $\boldsymbol{W}$ are drawn from a standard normal distribution $\boldsymbol{W}_{i,j} \sim \mathcal{N}(0,1)$, where $\boldsymbol{W}_{i,j}$ denotes the entry in row i, column j. Results are reported in Table 1.

Example 2*:* We generate latents using a factorial-design process. Specifically, we employ a 2^4 full factorial design for generating the synthetic data, resulting in $2^4 = 16$ modes. For each mode $k = 1, ...16$, we sample from a multivariate Gaussian distribution $\mathcal{N}(\boldsymbol{\mu}_k, \Sigma)$, where the covariance matrix Σ has entries $\Sigma_{i,j} = 0.5^{|i-j|}$. Here, the correlation parameter is set to

$\rho = 0.5$. The vector $\boldsymbol{\mu}_k$ is generated according to a 2^4 full-factorial design. The matrix Π is then defined as the corresponding 2^4 factorial-design matrix, as follows:

$$\Pi^\top = \begin{pmatrix} +1 & +1 & +1 & +1 & +1 & +1 & +1 & +1 & -1 & -1 & -1 & -1 & -1 & -1 & -1 & -1 \\ +1 & +1 & +1 & +1 & -1 & -1 & -1 & -1 & +1 & +1 & +1 & +1 & -1 & -1 & -1 & -1 \\ +1 & +1 & -1 & -1 & +1 & -1 & +1 & -1 & +1 & +1 & -1 & -1 & +1 & -1 & +1 & -1 \\ +1 & -1 & +1 & -1 & +1 & +1 & -1 & -1 & +1 & -1 & +1 & -1 & +1 & +1 & -1 & -1 \end{pmatrix}$$

The matrix Π controls the sign of each component of the latent mean vectors $\boldsymbol{\mu}_k$. The matrix $\boldsymbol{U}$ controls the coefficient of the vector $\boldsymbol{\mu}_k$: it is a 16×4 matrix where $\boldsymbol{U}_{i,j} \sim \text{Unif}(0,1)$. We denote by $\odot$ the Hadamard matrix product, so that for any conformable matrices A and B, it will have the following: $(\Pi \odot \boldsymbol{U})_{i,j} = \Pi_{i,j} \times \boldsymbol{U}_{i,j}$. Finally, each mean vector is given by $\boldsymbol{\mu}_k = (\Pi \odot \boldsymbol{U})_{i,\cdot}$.

Table 2. Performance comparison under simulation example 2.

			IP			MMD			PCT		
N	d	Method	$0.5\sqrt{N}$	$\sqrt{N}$	$2\sqrt{N}$	$0.5\sqrt{N}$	$\sqrt{N}$	$2\sqrt{N}$	$0.5\sqrt{N}$	$\sqrt{N}$	$2\sqrt{N}$
840	20	**KTCS**	0.160	0.118	0.097	0.074	0.032	0.008	0.451	0.683	0.830
		MED	0.095	0.077	0.075	0.070	0.052	0.038	0.566	0.813	0.951
		MMD-Critic	0.192	0.117	0.095	0.077	0.031	0.021	0.375	0.685	0.902
		SP	0.158	0.136	0.123	0.014	0.008	0.005	0.063	0.128	0.276
		SPARTAN	0.211	0.171	0.150	0.064	0.029	0.015	0.084	0.153	0.304
		Uniform	0.209	0.172	0.150	0.062	0.029	0.015	0.087	0.151	0.296
840	50	**KTCS**	0.116	0.078	0.061	0.080	0.037	0.013	0.517	0.778	0.899
		MED	0.076	0.048	0.040	0.072	0.052	0.038	0.558	0.803	0.946
		MMD-Critic	0.143	0.074	0.054	0.100	0.041	0.024	0.363	0.685	0.902
		SP	0.118	0.095	0.084	0.014	0.008	0.004	0.042	0.121	0.265
		SPARTAN	0.161	0.126	0.105	0.068	0.035	0.017	0.074	0.151	0.286
		Uniform	0.157	0.120	0.103	0.067	0.032	0.015	0.094	0.177	0.308
4200	20	**KTCS**	0.120	0.101	0.090	0.020	0.005	0.001	0.674	0.789	0.870
		MED	0.071	0.067	0.067	0.058	0.048	0.042	0.880	0.958	0.987
		MMD-Critic	0.111	0.090	0.104	0.033	0.024	0.020	0.820	0.945	0.977
		SP	0.137	0.124	0.115	0.006	0.004	0.003	0.165	0.345	0.566
		SPARTAN	0.174	0.152	0.138	0.028	0.013	0.007	0.182	0.323	0.553
		Uniform	0.171	0.151	0.139	0.027	0.014	0.007	0.177	0.330	0.554
4200	50	**KTCS**	0.081	0.066	0.058	0.028	0.009	0.003	0.773	0.868	0.925
		MED	0.043	0.035	0.034	0.057	0.047	0.041	0.870	0.957	0.987
		MMD-Critic	0.071	0.051	0.055	0.045	0.030	0.021	0.831	0.945	0.983
		SP	0.096	0.086	0.081	0.006	0.004	0.002	0.164	0.374	0.612
		SPARTAN	0.125	0.106	0.099	0.030	0.015	0.008	0.167	0.331	0.571
		Uniform	0.121	0.106	0.098	0.028	0.014	0.007	0.181	0.337	0.551

We randomly split the 16 modes into two parts, labeling one as Major, and the remaining half of them labeled as Minor, with a 20:1 ratio in sample sizes. In the first scenario ($N = 840$), each Major mode has 100 points and each Minor mode has 5 points. In the second scenario ($N = 4200$), each Major mode has 500 points and each Minor mode has 25 points. The transformation matrix $\boldsymbol{W}$ has entries $\boldsymbol{W}_{i,j} \sim \mathcal{N}(0,1)$. We generate final data in dimensions $d = 20$ and $d = 50$. Results are reported in Table 2, where our method achieves a balance between IP and MMD.

Example 3*:* We extend our 2^4 factorial design to a 2^5 full factorial design, with a total of 32 modes. 16 of modes are labeled as Major while the remaining 16 are labeled as Minor. The ratio of the number of points is 20:1. We examine two scenarios: one with $N = 1680$ points (100 per Major mode, 5 per Minor mode) and another with $N = 8400$ points (500 per Major mode, 25 per Minor mode). Results are reported in Table , where our method simultaneously achieves representativeness and coverage.

Table 3. Performance comparison under simulation example 3.

			IP			MMD			PCT		
N	d	Method	$0.5\sqrt{N}$	$\sqrt{N}$	$2\sqrt{N}$	$0.5\sqrt{N}$	$\sqrt{N}$	$2\sqrt{N}$	$0.5\sqrt{N}$	$\sqrt{N}$	$2\sqrt{N}$
1680	20	**KTCS**	0.103	0.075	0.059	0.049	0.021	0.008	0.311	0.510	0.696
		MED	0.061	0.046	0.042	0.054	0.037	0.026	0.502	0.708	0.857
		MMD-Critic	0.126	0.072	0.056	0.047	0.021	0.013	0.299	0.608	0.817
		SP	0.101	0.086	0.076	0.010	0.006	0.004	0.043	0.101	0.209
		SPARTAN	0.138	0.110	0.093	0.046	0.024	0.011	0.056	0.121	0.220
		Uniform	0.137	0.108	0.092	0.045	0.022	0.011	0.063	0.128	0.236
1680	50	**KTCS**	0.073	0.047	0.035	0.053	0.025	0.011	0.376	0.614	0.797
		MED	0.051	0.030	0.023	0.048	0.031	0.021	0.507	0.716	0.862
		MMD-Critic	0.092	0.045	0.031	0.060	0.025	0.014	0.296	0.611	0.817
		SP	0.064	0.051	0.043	0.012	0.006	0.004	0.039	0.100	0.207
		SPARTAN	0.094	0.069	0.056	0.048	0.024	0.012	0.062	0.119	0.219
		Uniform	0.094	0.069	0.055	0.047	0.023	0.011	0.057	0.118	0.218
8400	20	**KTCS**	0.075	0.061	0.052	0.017	0.006	0.002	0.514	0.665	0.770
		MED	0.041	0.036	0.034	0.043	0.034	0.029	0.749	0.852	0.907
		MMD-Critic	0.068	0.052	0.058	0.022	0.016	0.011	0.707	0.871	0.943
		SP	0.086	0.076	0.069	0.005	0.003	0.002	0.114	0.237	0.424
		SPARTAN	0.109	0.091	0.081	0.020	0.010	0.005	0.128	0.246	0.430
		Uniform	0.108	0.092	0.082	0.020	0.010	0.005	0.131	0.242	0.424
8400	50	**KTCS**	0.048	0.037	0.031	0.022	0.009	0.003	0.609	0.763	0.847
		MED	0.027	0.020	0.017	0.034	0.027	0.023	0.751	0.847	0.899
		MMD-Critic	0.042	0.028	0.025	0.026	0.017	0.012	0.707	0.864	0.946
		SP	0.051	0.044	0.041	0.005	0.003	0.002	0.112	0.245	0.442
		SPARTAN	0.068	0.057	0.050	0.022	0.011	0.005	0.127	0.243	0.410
		Uniform	0.069	0.057	0.050	0.021	0.010	0.005	0.124	0.245	0.442

In conclusion, our simulations across multiple data-generation scenarios consistently demonstrate that the KTCS algorithm balances representativeness and coverage. Compared with state-of-the-art methods, KTCS achieves larger IP values and lower MMD scores while capturing a high proportion of minor modes. These findings confirm that KTCS not only selects representative samples from complex data but also successfully covers safety-critical and corner cases far from the distribution's center. Its performance advantages persist across different sample sizes and dimensions, reflecting the robustness and generalizability of our approach.

Additional Results

Example of Test Case

Figure 1 shows an example raw test case from the SHRP2 NDS dataset. Panels 1 (a)-(c) present the subject vehicle's three-axis accelerations, longitudinal acceleration (*accx*), lateral acceleration (*accy*), and vertical acceleration (*accz*), as driving dynamics over a 15-second interval. Panel (d) then shows the resulting speed profile, increasing from approximately 1 m/s to about 18 m/s,

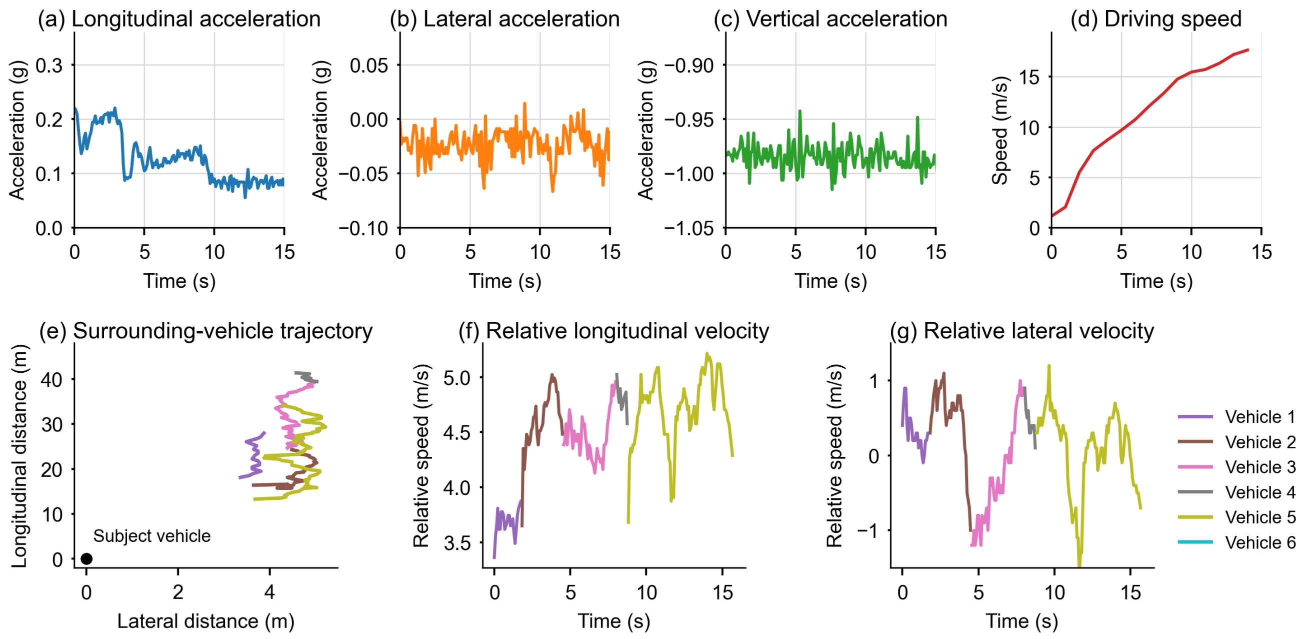

Figure 1. A raw test case sample from the candidate pool. (a–c) Time series of the subject vehicle's longitudinal (*accx*), lateral (*accy*), and vertical (*accz*) accelerations over 15 seconds. (d) Subject vehicle speed profile. (e) Trajectories of six surrounding vehicles detected by radar. (f) Relative longitudinal speeds between the subject vehicle and each surrounding vehicle. (g) Relative lateral speeds between the subject vehicle and each surrounding vehicle.

which corresponds to the positive acceleration observed in panels (a)-(c). Panels 1 (e)-(g) characterize the surrounding traffic detected by the radar sensors: (e) shows the trajectories of six surrounding vehicles over the same 15-second window, with each color representing a different vehicle, while (f) and (g) display the relative longitudinal and lateral speeds, respectively, between the subject vehicle and each of the six surrounding vehicles.

Determining the Number of Test Cases

In determining the number of selected cases, Theorem 3 demonstrates that choosing $M = \mathscr{O}(\sqrt{N})$ achieves desirable convergence properties: the M selected cases closely approximate the distribution of the full set of N candidates test cases. The setting $M = 0.5\sqrt{N}$ achieves satisfactory performance in both representativeness and coverage. Industrial-scale ADS safety evaluations typically conduct on the order of one hundred test runs due to real-world budget and time constraints. For instance,[8] executed 117 test cases. Balancing this practical benchmark with our theoretical guidance, we choose $M = 0.5\sqrt{N}$, which is 118 selected test cases.

All Selected Test Cases

The 48 extracted features, along with their calculation formula and engineering interpretations, are presented in Table 4. Figures 2 to Figure 7 present the 118 cases using radar plots. Each plot is annotated with its attention-generated weights, case ID, and

Table 4. All extracted features and their associated calculation formula and descriptions

Driving Data	Feature	Formula	Engineering Interpretation
X-Axis Acceleration	Initiation	$accx\text{-}init = a_{x,1}$	Longitudinal acceleration at initial time
	Mean	$accx\text{-}mean = \frac{1}{T}\sum_{t=1}^{T} a_{x,t}$	Average of the longitudinal acceleration
	Maximum	$accx\text{-}max = \max[a_{x,1},...a_{x,T}]$	Maximum of longitudinal acceleration
	Minimum	$accx\text{-}min = \min[a_{x,1},...a_{x,T}]$	Minimum of longitudinal acceleration
	Std Dev	$accx\text{-}std = \mathrm{SD}[a_{x,1},...a_{x,T}]$	Level of volatility in longitudinal acceleration
	Skewness	$accx\text{-}skew = \mathrm{Skew}[a_{x,1},...a_{x,T}]$	Skewness of the longitudinal acceleration
	Kurtosis	$accx\text{-}kurt = \mathrm{Kurt}[a_{x,1},...a_{x,T}]$	Kurtosis of the longitudinal acceleration
Y-Axis Acceleration	Initiation	$accy\text{-}init = a_{y,1}$	Lateral acceleration at initial time
	Mean	$accy\text{-}mean = \frac{1}{T}\sum_{t=1}^{T} a_{y,t}$	Average of the lateral acceleration
	Maximum	$accy\text{-}max = \max[a_{y,1},...a_{y,T}]$	Maximum of lateral acceleration
	Minimum	$accy\text{-}min = \min[a_{y,1},...a_{y,T}]$	Minimum of lateral acceleration
	Std Dev	$accy\text{-}std = \mathrm{SD}[a_{y,1},...a_{y,T}]$	Standard deviation of lateral acceleration
	Skewness	$accy\text{-}skew = \mathrm{Skew}[a_{y,1},...a_{y,T}]$	Skewness of the lateral acceleration
	Kurtosis	$accy\text{-}kurt = \mathrm{Kurt}[a_{y,1},...a_{y,T}]$	Kurtosis of the lateral acceleration
Z-Axis Acceleration	Initiation	$accz\text{-}init = a_{z,1}$	Horizontal acceleration at initial time
	Mean	$accz\text{-}mean = \frac{1}{T}\sum_{t=1}^{T} a_{z,t}$	Average of the horizontal acceleration
	Maximum	$accz\text{-}max = \max[a_{z,1},...a_{z,T}]$	Maximum of horizontal acceleration
	Minimum	$accz\text{-}min = \min[a_{z,1},...a_{z,T}]$	Minimum of horizontal acceleration
	Std Dev	$accz\text{-}std = \mathrm{SD}[a_{z,1},...a_{z,T}]$	Standard deviation of horizontal acceleration
	Skewness	$accz\text{-}skew = \mathrm{Skew}[a_{z,1},...a_{z,T}]$	Skewness of the horizontal acceleration
	Kurtosis	$accz\text{-}kurt = \mathrm{Kurt}[a_{z,1},...a_{z,T}]$	Kurtosis of the horizontal acceleration
Driving Speed	Initiation	$speed\text{-}init = v_1$	Driving speed at initial time of the case
	Mean	$speed\text{-}mean = \frac{1}{T}\sum_{t=1}^{T} v_t$	Average of speed within the case
	Maximum	$speed\text{-}max = \max[v_1,...v_T]$	Maximum of speed within the case
	Minimum	$speed\text{-}min = \min[v_1,...v_T]$	Minimum of speed within the case
	Std Dev	$speed\text{-}std = \mathrm{SD}[v_1,...v_T]$	Standard deviation of speed within the case
	Skewness	$speed\text{-}skew = \mathrm{Skew}[v_1,...v_T]$	Skewness of speed within the case
	Kurtosis	$speed\text{-}kurt = \mathrm{Kurt}[v_1,...v_T]$	Kurtosis of speed within the case
Relative X-Axis Position	Initiation	$xpos\text{-}init = \Delta s_{x,1}$	Relative longitudinal distance at initial time
	Mean	$xpos\text{-}mean = \frac{1}{T}\sum_{t=1}^{T} \Delta s_{x,t}$	Average of relative longitudinal distance
	Maximum	$xpos\text{-}max = \max[\Delta s_{x,1},...\Delta s_{x,T}]$	Largest relative longitudinal distance
	Minimum	$xpos\text{-}min = \min[\Delta s_{x,1},...\Delta s_{x,T}]$	Closest relative longitudinal distance
	Std Dev	$xpos\text{-}std = \mathrm{SD}[\Delta s_{x,1},...\Delta s_{x,T}]$	Level of interactions in relative longitudinal position
Relative Y-Axis Position	Initiation	$ypos\text{-}init = \Delta s_{y,1}$	Relative lateral distance at initial time
	Mean	$ypos\text{-}mean = \frac{1}{T}\sum_{t=1}^{T} \Delta s_{y,t}$	Average of relative lateral distance
	Maximum	$ypos\text{-}max = \max[\Delta s_{y,1},...\Delta s_{y,t}]$	Maximum of relative lateral distance
	Minimum	$ypos\text{-}min = \min[\Delta s_{y,1},...\Delta s_{y,T}]$	Minimum of relative lateral distance
	Std Dev	$ypos\text{-}std = \mathrm{SD}[\Delta s_{y,1},...\Delta s_{y,T}]$	Level of interactions in relative lateral position
Relative X-Axis Velocity	Initiation	$xvel\text{-}init = \Delta v_{x,1}$	Relative longitudinal velocity at initial time
	Mean	$xvel\text{-}mean = \frac{1}{T}\sum_{t=1}^{T} \Delta v_{x,t}$	Average of longitudinal velocity
	Maximum	$xvel\text{-}max = \max[\Delta v_{x,1},...\Delta v_{x,T}]$	Maximum of longitudinal velocity
	Minimum	$xvel\text{-}min = \min[\Delta v_{x,1},...\Delta v_{x,T}]$	Minimum of longitudinal velocity
	Std Dev	$xvel\text{-}std = \mathrm{SD}[\Delta v_{x,1},...\Delta v_{x,T}]$	Level of interactions in relative longitudinal velocity
Relative Y-Axis Velocity	Initiation	$yvel\text{-}init = \Delta v_{y,1}$	Relative lateral velocity at initial time
	Mean	$yvel\text{-}mean = \frac{1}{T}\sum_{t=1}^{T} \Delta v_{y,t}$	Average of relative lateral velocity
	Maximum	$yvel\text{-}max = \max[\Delta v_{y,1},...\Delta v_{y,T}]$	Maximum of relative lateral velocity
	Minimum	$yvel\text{-}min = \min[\Delta v_{y,1},...\Delta v_{y,T}]$	Minimum of relative lateral velocity
	Std Dev	$yvel\text{-}std = \mathrm{SD}[\Delta v_{y,1},...\Delta v_{y,T}]$	Level of interactions in relative lateral velocity

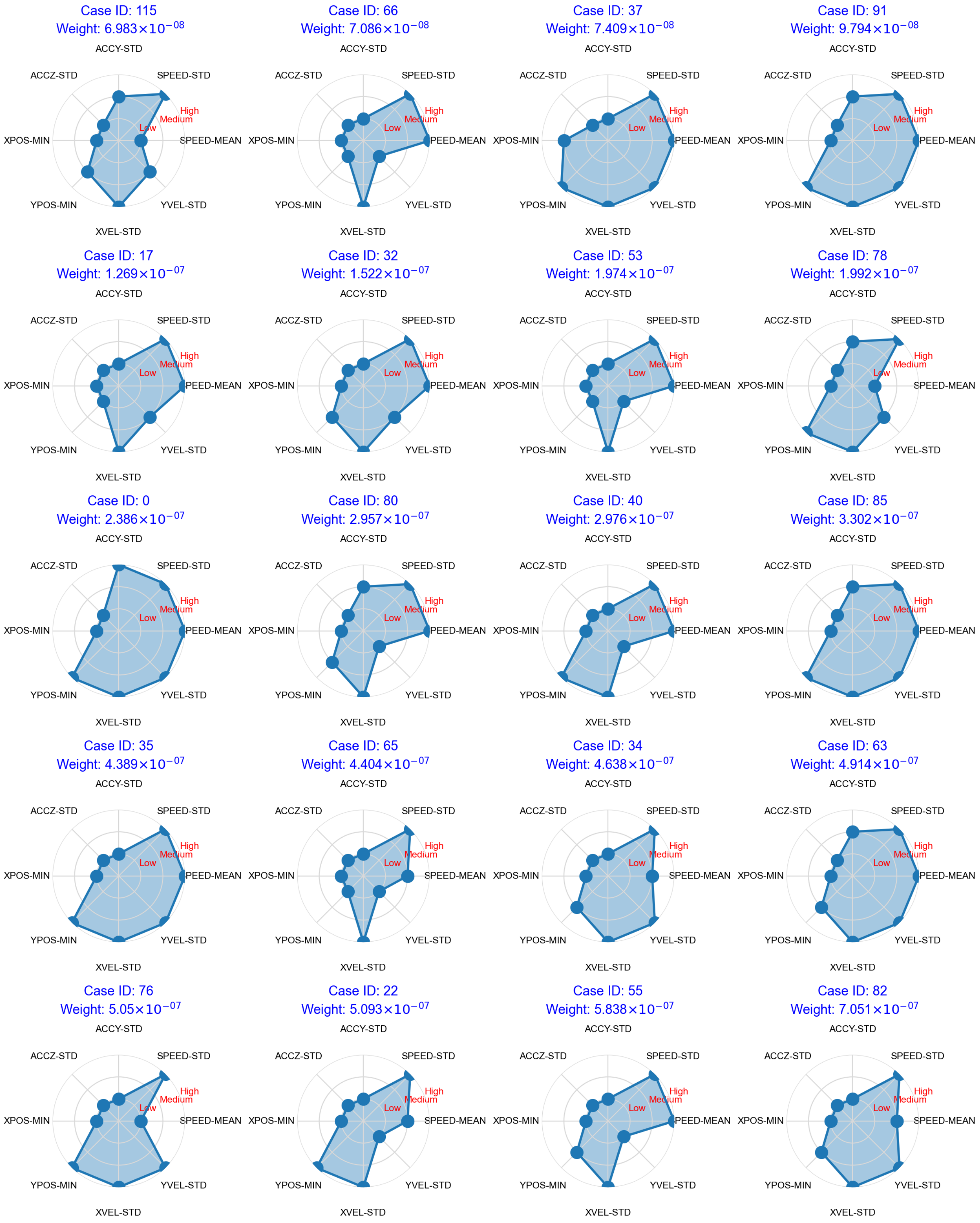

Figure 2. Part 1 of the 118 selected cases with corresponding weight and selected features.

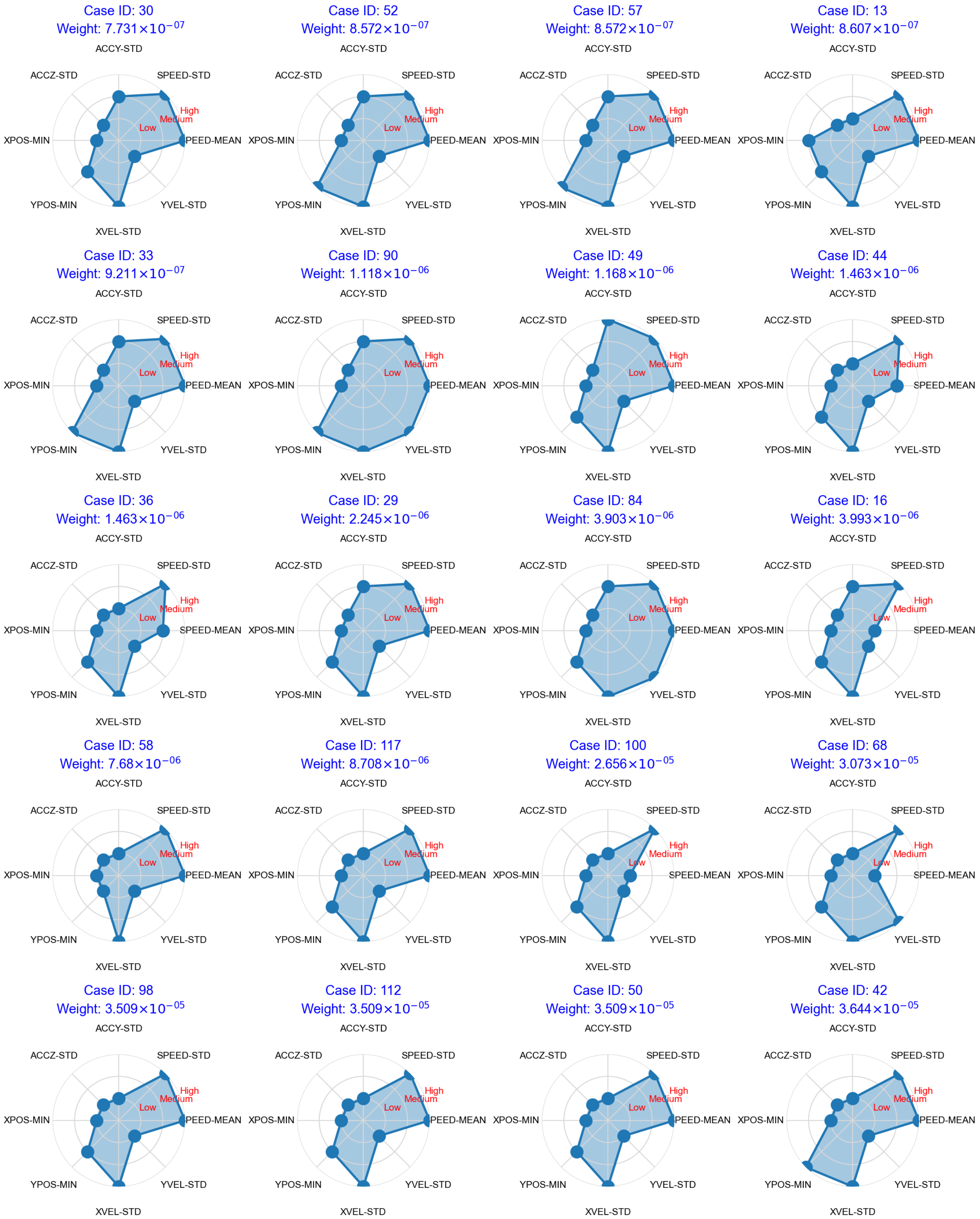

Figure 3. Part 2 of the 118 selected cases with corresponding weight and selected features.

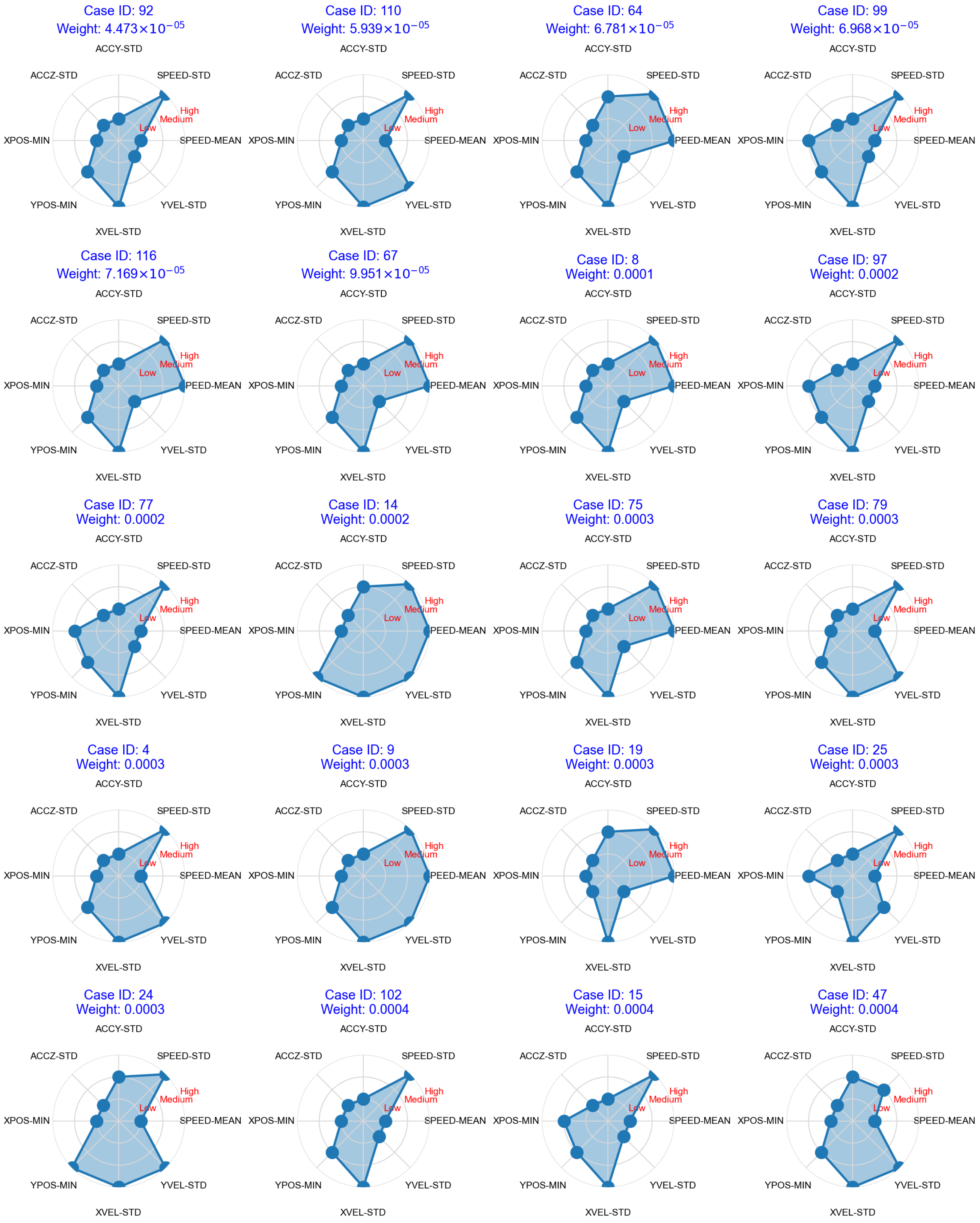

Figure 4. Part 3 of the 118 selected cases with corresponding weight and selected features.

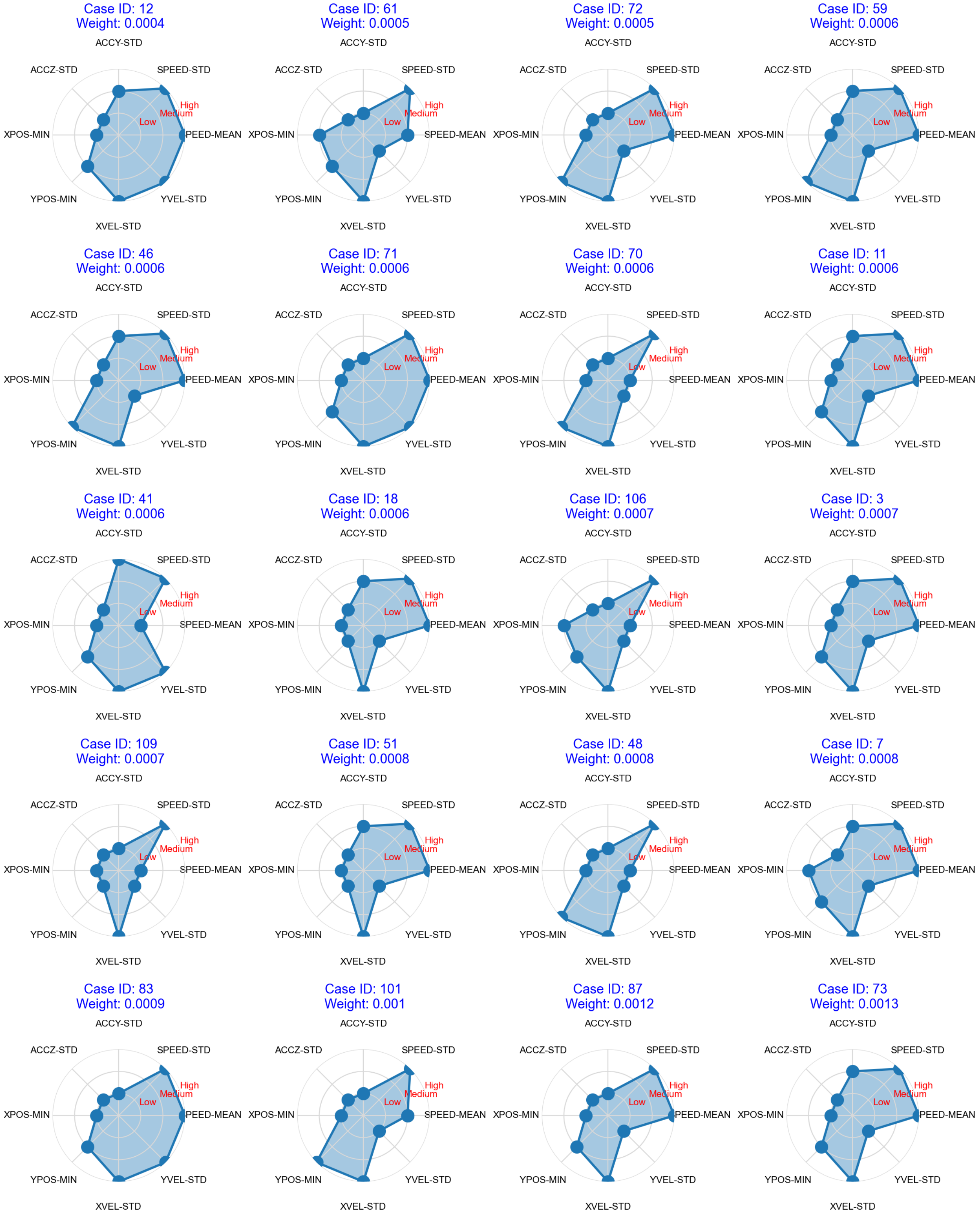

Figure 5. Part 4 of the 118 selected cases with corresponding weight and selected features.

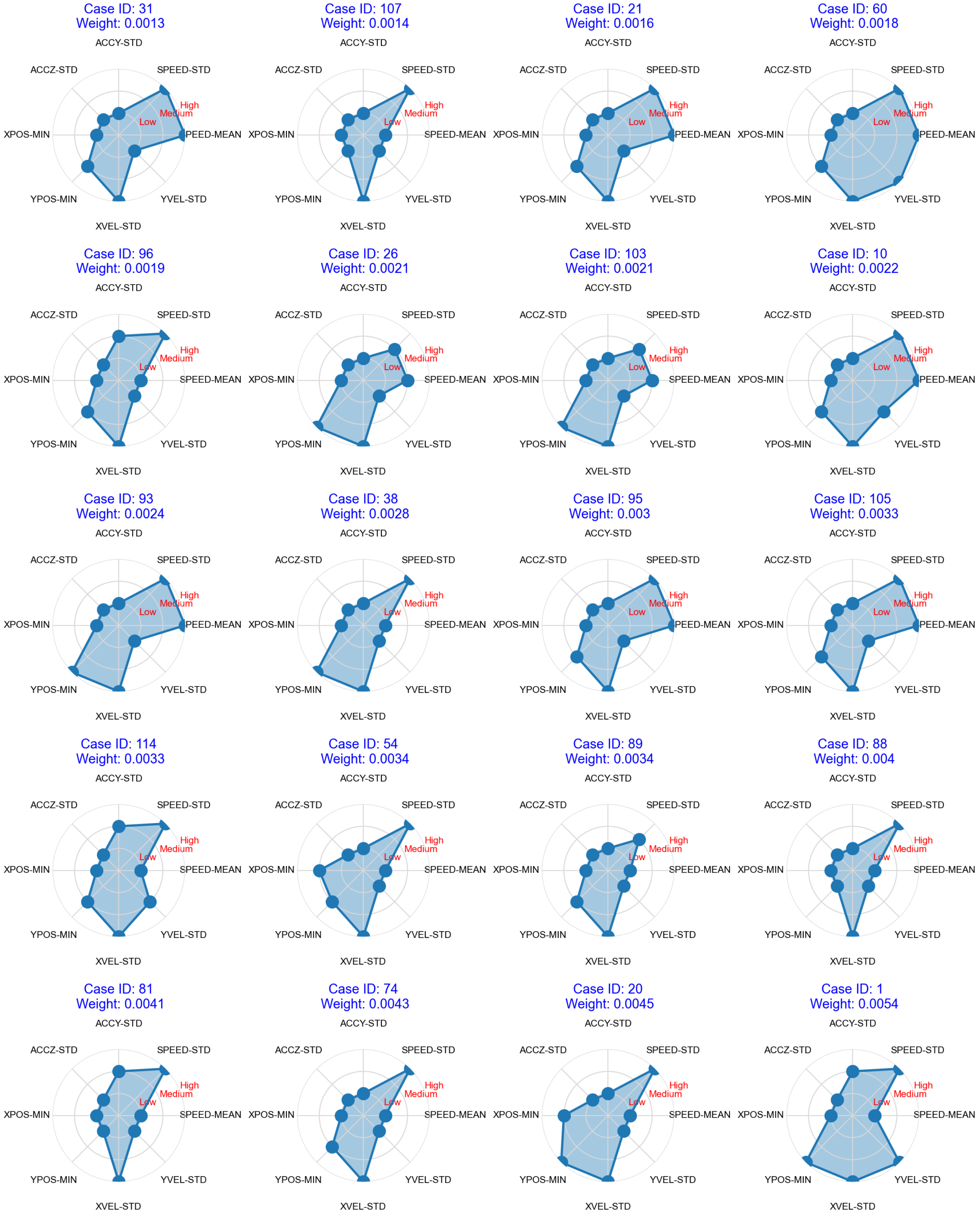

Figure 6. Part 5 of the 118 selected cases with corresponding weight and selected features.

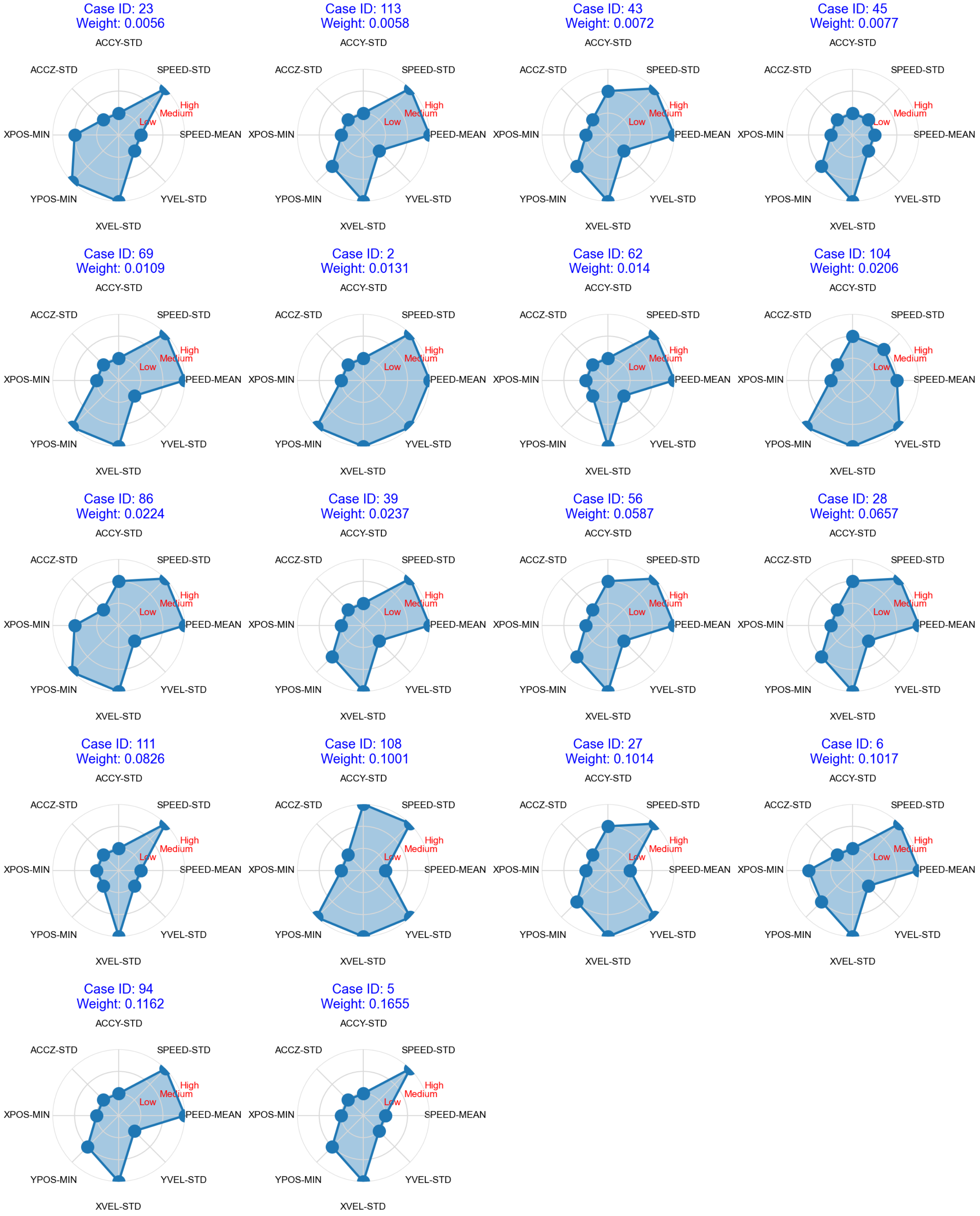

Figure 7. Part 6 of the 118 selected cases with corresponding weight and selected features.

descriptive metrics for the selected features.

Figure 7 Explanation

In Figure 7 of the manuscript, the percentages are calculated based on each case's relative distance to the centroid of the candidate pool in multidimensional space. First, we compute the centroid as $\bar{\boldsymbol{x}} = \sum_{i=1}^{N} \boldsymbol{x}_i$. Second, for each case $\boldsymbol{x}_i$, we calculate its squared Euclidean distance to the centroid as $D_i = \|\boldsymbol{x}_i - \bar{\boldsymbol{x}}\|_2^2$, yielding distances $D_1, \ldots, D_N$. We then determine the p-th quantile threshold q_p of the set D_i. Third, for each selected case z_i, we compute $D_i^s = \|\boldsymbol{z}_i - \bar{\boldsymbol{x}}\|_2^2$. Finally, we count how many of the M selected cases satisfy $D_i^s > q_p$.

Ablation Study

We conduct the following ablation studies to validate the role of the λ-weights in the proposed KTCS algorithm. The weights $\lambda_1, \ldots, \lambda_M$ realign the distributions of the M selected cases with that of the original N-sized candidate test case pool. To quantitatively assess the contribution of Step 2 to the overall approximation quality, we consider two additional variants of the KTCS algorithm: (1) No Alignment: No distributional alignment is applied; each case is equally weighted. (2) w-weights Alignment: The λ-weights are replaced with the w-weights derived in Step 1 of the KTCS algorithm. The performances of the MMD, ADD and H-dist metrics are reported in Table 5.

To assess whether our method can capture the true shape of high-dimensional distributions, particularly different moment information, we measure discrepancies at three moment levels for each setting: (1) Mean Discrepancy (Mean Dis): Measures the difference in mean values, calculated as $\|\mu_M - \mu_N\|_2$, where μ_M and μ_N are the mean vectors of the M selected cases and the N-sized candidate pool, respectively; lower values indicate closer means. (2) Variance Discrepancy (Var Dis): Measures the difference in variance across dimensions, calculated as $\frac{1}{d}\sum_{i=1}^{d} |\sigma_{M,i}^2 - \sigma_{N,i}^2|$, where $\sigma_{M,i}^2$ and $\sigma_{N,i}^2$ are variances along the i-th dimension; lower values indicate more similar variances. (3) Covariance Discrepancy (Cov Dis): Measures the difference in covariance structures, calculated as $\|\Sigma_M - \Sigma_N\|_F / \|\Sigma_N\|_F)$, where Σ_M and Σ_N are the covariance matrices constructed from the M selected cases and the candidate pool, respectively, and $\|\cdot\|_F)$ denotes the Frobenius norm; lower values indicate more similar covariance structures. The reports are presented in Table 5.

Table 5 shows that Step 2 of the KTCS algorithm decreases distributional distances (MMD, ADD, H-Dist) by an order of magnitude and substantially improves moment-matching accuracy.

Table 5. Ablation study of the KTCS algorithm.

Metric	λ-weights Alignment	No Alignment	w-weights Alignment
MMD ↓	0.020	0.286	0.382
ADD ↓	0.018	0.317	0.442
H-Dist ↓	1.007	3.877	5.079
Mean dis ↓	0.091	0.644	1.559
Var Dis ↓	0.003	0.030	0.042
Cov Dis ↓	0.035	0.255	0.523

Note: ↓ indicates that lower values are better. H-dist is an empirical distance measure that can take negative values[3].

Lemmas and Proofs

Lemmas

Lemma 1*:* Let $X_1,\dots,X_n$ be i.i.d. random variables on Hilbert space with the condition that $\sup_{i=1,\dots,n}\|X_i\|_{\mathscr{H}} \leq A$ for some real number $A>0$. Then, for any $\delta \in (0,1)$, the following holds with probability at least $1-\delta$ that

$$\Pr\left(\|\frac{1}{n}\sum_{i=1}^{n} X_i\|_{\mathscr{H}} \leq A\frac{\sqrt{2\log(2/\delta)}}{\sqrt{n}}\right) \geq 1-\delta \tag{2}$$

Lemma 2*:* Let $X_1,\dots,X_n$ be i.i.d. random variables in a Hilbert space $(\mathscr{H},\|\cdot\|)$ such that - $\forall i\in[n], \mathbb{E}X_i=\mu$, - $\exists\sigma>0, \exists H>0, \forall i\in[n], \forall p\geq 2, \mathbb{E}\|X_i-\mu\|^p \leq \frac{1}{2}p!\sigma^2 H^{p-2}$. Then, for any $\delta\in[0,1]$, we have with probability at least $1-\delta$,

$$\|\frac{1}{n}\sum_{i=1}^{n} X_i-\mu\|_{\mathscr{H}} \leq \frac{2H\log(2/\delta)}{n}+\sqrt{\frac{2\sigma^2\log(2/\delta)}{n}} \tag{3}$$

Lemma 1 rely on the Theorem 3.5 in[9], and Lemma 2 rely on Theorem 3.3.4 of[10].[11] and[12] use similar approaches.

Lemma 3 (McDiamrd inequality)*:* Suppose $X_1,\dots,X_N$ in the domain of $\mathscr{X}$ are independent random variables. For any function $f:\mathscr{X}^N\to\mathbb{R}^N$, there exists a constant $c_i>0$, such that $\left|f(X_1,\dots,X_i,\dots,X_N)-f(X_1,\dots,X_i',\dots,X_N\right|\leq c_i$, then the following holds:

$$\Pr\left(\left|f(X_1,\dots,X_N)-\mathbb{E}[f(X_1,\dots,X_N)]\right|\geq\varepsilon\right)\leq 2\exp\left(-\frac{2\varepsilon^2}{\sum_{i=1}^{N}c_i^2}\right) \tag{4}$$

where ε is some constant here.

Lemma 4 (Hoeffding inequality)*:* Suppose $X_1,\dots,X_n$ are independent random variables satisfying that $\mathbb{E}[X_i]=0$ and $a_i\leq X_i\leq b_i$

for all i. If $\varepsilon > 0$ and for all $t > 0$, then:

$$\Pr\left[\frac{1}{n}\sum_{i=1}^{n} X_i \geq \varepsilon\right] \leq e^{-nt\varepsilon}\prod_{i=1}^{n} e^{t^2(b_i-a_i)^2/8} \tag{5}$$

where ε is some constant here.

Proofs

Theorem 1*:* Define $\mathrm{IP}_{\boldsymbol{z}}$ as the information potential with the selected points through Pareto order sampling. When the size N goes sufficiently large, the following holds:

$$\mathrm{IP}_{\boldsymbol{z}} \to \mathscr{E}_X(\theta_{\mathrm{opt}}) \quad \text{as} \quad N \to \infty \quad \text{where} \quad \mathscr{E}_X(\theta_{\mathrm{opt}}) = \sum_{i=1}^{N}\sum_{j\neq i}^{N} w_{\theta_{\mathrm{opt}}}(\boldsymbol{x}_i) w_{\theta_{\mathrm{opt}}}(\boldsymbol{x}_j)\mathscr{K}(\boldsymbol{x}_i,\boldsymbol{x}_j) \tag{6}$$

Proof: For simplicity, we define $w_{\theta_{\mathrm{opt}}}(\boldsymbol{x}_i) = \psi_i$. Based on this, we can have the follows:

$$\mathscr{E}_X(\theta_{\mathrm{opt}}) = \sum_{i=1}^{N}\sum_{j\neq i}^{N} w_{\theta_{\mathrm{opt}}}(\boldsymbol{x}_i) w_{\theta_{\mathrm{opt}}}(\boldsymbol{x}_j)\mathscr{K}(\boldsymbol{x}_i,\boldsymbol{x}_j) = \sum_{i=1}^{N}\sum_{j\neq i}^{N} \psi_i\psi_j\mathscr{K}(\boldsymbol{x}_i,\boldsymbol{x}_j) \tag{7}$$

Similar to the inclusion measure in[13], we define π_i as the factual importance measure approximated through Pareto-order sampling and ψ_i as the target importance measure. To investigate the asymptotic behaviors of $\widehat{\mathrm{IP}}_{\boldsymbol{z}}$, we decompose its difference from $\mathscr{E}_X(\theta_{\mathrm{opt}})$ as follows:

$$\widehat{\mathrm{IP}}_{\boldsymbol{z}} - \mathscr{E}_X(\theta_{\mathrm{opt}}) = \underbrace{\widehat{\mathrm{IP}}_{\boldsymbol{z}} - \sum_{i=1}^{N}\sum_{j\neq i}^{N} \pi_i\pi_j\mathscr{K}(\boldsymbol{x}_i,\boldsymbol{x}_j)}_{T_1 \text{ Term}} + \underbrace{\sum_{i=1}^{N}\sum_{j\neq i}^{N} \pi_i\pi_j\mathscr{K}(\boldsymbol{x}_i,\boldsymbol{x}_j) - \mathscr{E}_X(\theta)}_{T_2 \text{ Term}} \tag{8}$$

Thus, investigating the asymptotic behaviors of $\widehat{\mathrm{IP}}_{\boldsymbol{z}} - \mathscr{E}_X(\theta_{\mathrm{opt}})$ is equivalent to verifying the T_1 and T_2 terms individually. First, we examine the T_1 term. We define $I(\boldsymbol{x}_i)$ as an indicator of whether $\boldsymbol{x}_i$ is included in the Pareto-order sampling. Using this, the

T_1 term can be decomposed as:

$$\begin{aligned} T_1 =& \frac{1}{M(M-1)} \sum_{t=1}^{M} \sum_{s=1, s\neq t}^{M} \mathscr{K}(\boldsymbol{z}_t, \boldsymbol{z}_s) - \sum_{i=1}^{N} \sum_{j\neq i}^{N} \pi_i \pi_j \mathscr{K}(\boldsymbol{x}_i, \boldsymbol{x}_j) \\ =& \sum_{t=1}^{M} \sum_{s=1, s\neq t}^{M} \frac{1}{M(M-1)} \mathscr{K}(\boldsymbol{z}_t, \boldsymbol{z}_s) - \sum_{i=1}^{N} \sum_{j\neq i}^{N} \pi_i \pi_j \mathscr{K}(\boldsymbol{x}_i, \boldsymbol{x}_j) \\ =& \sum_{t=1}^{N} \sum_{s=1, s\neq t}^{N} \frac{I(\boldsymbol{x}_i) I(\boldsymbol{x}_j)}{M(M-1)} \mathscr{K}(\boldsymbol{x}_i, \boldsymbol{x}_j) - \sum_{i=1}^{N} \sum_{j\neq i}^{N} \pi_i \pi_j \mathscr{K}(\boldsymbol{x}_i, \boldsymbol{x}_j) \\ =& \sum_{t=1}^{N} \sum_{s=1, s\neq t}^{N} \left[\frac{I(\boldsymbol{x}_i) I(\boldsymbol{x}_j)}{M(M-1)} - \pi_i \pi_j \right] \mathscr{K}(\boldsymbol{x}_i, \boldsymbol{x}_j) \end{aligned} \tag{9}$$

Since $\mathbb{E}[I(\boldsymbol{x}_i)] = M\pi_i$, it follows that:

$$\mathbb{E}[I(\boldsymbol{x}_i) I(\boldsymbol{x}_j)] = \frac{M(M-1)}{N(N-1)} \times N(N-1)\pi_i \pi_j = \frac{M(M-1)}{N(N-1)} \pi_i' \pi_j' \tag{10}$$

Thus, we obtain the following equations:

$$\mathbb{E}\left[\frac{N(N-1)}{M(M-1)} I(\boldsymbol{x}_i) I(\boldsymbol{x}_j) - \pi_i' \pi_j' \right] = 0 \tag{11}$$

From this equation, it follows that:

$$\frac{I(\boldsymbol{x}_i) I(\boldsymbol{x}_j)}{M(M-1)} - \pi_i \pi_j = \frac{1}{N(N-1)} \left[\frac{N(N-1)}{M(M-1)} I(\boldsymbol{x}_i) I(\boldsymbol{x}_j) - \pi_i' \pi_j' \right] \tag{12}$$

Substituting into Equation 12, the T_1 term simplifies to:

$$\begin{aligned} T_1 =& \frac{1}{N(N-1)} \sum_{t=1}^{N} \sum_{s=1, s\neq t}^{N} \left[\frac{N(N-1)}{M(M-1)} I(\boldsymbol{x}_i) I(\boldsymbol{x}_j) - \pi_i' \pi_j' \right] \\ =& \frac{1}{N(N-1)} \sum_{i\neq j} \left[\frac{N(N-1)}{M(M-1)} I(\boldsymbol{x}_i) I(\boldsymbol{x}_j) - \pi_i' \pi_j' \right] \end{aligned} \tag{13}$$

We set that $\left[\frac{N(N-1)}{M(M-1)} I(\boldsymbol{x}_i) I(\boldsymbol{x}_j) - \pi_i' \pi_j' \right] \in [-u, u]$. For simplicity, let $N' = N(N-1)$. Applying Lemma 4, we obtain the following concentration inequality:

$$\Pr\left(\frac{1}{N(N-1)} \sum_{i\neq j} \left[\frac{N(N-1)}{M(M-1)} I(\boldsymbol{x}_i) I(\boldsymbol{x}_j) - \pi_i' \pi_j' \right] \geq \varepsilon \right) \leq \exp\left(-N' t \varepsilon + \frac{u^2 t^2 N'}{2} \right) \tag{14}$$

If we let $t = \varepsilon/u^2$ and $\varepsilon = \sqrt{\frac{-2u^2 \log \delta}{N'}}, \delta \in (0,1)$. Then, with probability at least $1-\delta$, we obtain the following bound:

$$\frac{1}{N(N-1)} \sum_{i \neq j} \left[\frac{N(N-1)}{M(M-1)} I(\boldsymbol{x}_i) I(\boldsymbol{x}_j) - \pi_i' \pi_j' \right] \leq \sqrt{\frac{-2u^2 \log \delta}{N'}} \tag{15}$$

As long as $u < N$, then $\sqrt{\frac{-2u^2 \log \delta}{N'}} \to 0$ when $N \to 0$. Since π_i is the factual importance measure from Pareto-order sampling with $\pi > 0$ and $\sum_{i=1}^{N} \pi_i = 1$, we have $\pi_i = o(1/N)$. Consequently, $\pi_i' \pi_j' = N(N-1)\pi_i \pi_j < N$, so the condition that $u < N$ is satisfied. Therefore, as N grows sufficiently large, $T_1 \to 0$.

For the T_2 term, applying equation (1.25) from[13], we have:

$$\pi_i / \psi_i \to 1 \quad \text{as} \quad N \to \infty, \quad i = 1, \ldots N \tag{16}$$

Based on the continuous mapping theorem, the following holds that:

$$\frac{\sum_{i=1}^{N} \sum_{j \neq i}^{N} \pi_i \pi_j \mathscr{K}(\boldsymbol{x}_i, \boldsymbol{x}_j)}{\sum_{i=1}^{N} \sum_{j \neq i}^{N} \psi_i \psi_j \mathscr{K}(\boldsymbol{x}_i, \boldsymbol{x}_j)} \to 1 \quad \text{as} \quad N \to \infty \tag{17}$$

which implies $T_2 \to 0$ as $N \to \infty$. Combining this with the asymptotic behaviors of the T_1 term, we have:

$$\widehat{\mathrm{IP}}_{\boldsymbol{z}} / \mathscr{E}_X(\theta) \to 1 \quad \text{as} \quad N \to \infty \tag{18}$$

This completes the proof.

Theorem 2: Assume that the parameter θ falls on the p dimensional unit ball (i.e. $\theta \in \mathscr{B}^p$) ε is some constant, and $w_\theta(\boldsymbol{x}) \leq s$. With at least a probability of $1-\delta$:

$$\mathscr{E}(\theta^*) - \mathscr{E}_X(\theta_{\text{opt}}) \leq R_N + \frac{s^2 K}{\sqrt{N}} \sqrt{2 \log(2/\delta) + 2p \log\left(1 + \frac{2}{\varepsilon}\right)} \tag{19}$$

where $\theta^* = \underset{\theta \in \mathscr{B}^p}{\operatorname{argmin}}\, \mathscr{E}(\theta)$, $\mathscr{E}(\theta) = \int_{\boldsymbol{x} \in \mathbb{X}} \int_{\boldsymbol{x}' \in \mathbb{X}} \mathscr{K}(\boldsymbol{x}, \boldsymbol{x}') w_\theta(\boldsymbol{x}) w_\theta(\boldsymbol{x}') d\mathbb{P}(\boldsymbol{x}, \boldsymbol{x}')$ and R_N is the empirical Rademacher complexity.

Proof: We first define the population version of $\mathscr{E}(\theta)$, denoted $\mathscr{E}_X(\theta)$, as follows:

$$\mathscr{E}(\theta) = \int_{\boldsymbol{x} \in \mathscr{X}} \int_{\boldsymbol{x}' \in \mathscr{X}} \mathscr{K}(\boldsymbol{x}, \boldsymbol{x}') w_\theta(\boldsymbol{x}) w_\theta(\boldsymbol{x}') d\mathbb{P}(\boldsymbol{x}, \boldsymbol{x}') \tag{20}$$

where $\theta^* = \underset{\theta \in \mathscr{B}^p}{\operatorname{argmin}} \, \mathscr{E}(\theta)$. Then we take the differences between these two functions defined as $\mathscr{E}(\theta) - \mathscr{E}_X(\theta)$ and such differences can be bounded as following:

$$\mathscr{E}(\theta) - \mathscr{E}_X(\theta) \leq \sup_{\theta \in \mathscr{B}^p} \left\{ \mathscr{E}(\theta) - \mathscr{E}_X(\theta) \right\} \tag{21}$$

Given that $\mathscr{E}_X(\theta)$ is computed from the sample $\boldsymbol{x}_1, \ldots, \boldsymbol{x}_N$, suppose we replace one element $\boldsymbol{x}_i$ with an alternative value $\boldsymbol{x}_i'$. Using the resulting sequence $(\boldsymbol{x}_1, \ldots, \boldsymbol{x}_i', \ldots, \boldsymbol{x}_N)$, we can similarly compute $\mathscr{E}X'(\theta)$. For simplicity, we define $\Lambda(X) = \mathscr{E}(\theta) - \mathscr{E}_X(\theta)$ and similarly $\Lambda(X') = \mathscr{E}(\theta) - \mathscr{E}_{X'}(\theta)$. We further define $\eta_\theta(\boldsymbol{x}_i, \boldsymbol{x}_j) = w_\theta(\boldsymbol{x}_i) w_\theta(\boldsymbol{x}_j) \mathscr{K}(\boldsymbol{x}_i, \boldsymbol{x}_j)$

Accordingly, we can compute the difference between $\Lambda(X)$ and $\Lambda(X')$ as follows:

$$\begin{aligned} \left|\Lambda(X) - \Lambda(X')\right| &= \sup_{\theta \in \mathscr{B}^p} \left|\mathscr{E}_X(\theta) - \mathscr{E}_{X'}(\theta)\right| \\ &= \sup_{\theta \in \mathscr{B}^p} \sum_{j \neq k} \left|\eta_\theta(\boldsymbol{x}_i, \boldsymbol{x}_j) - \eta_\theta(\boldsymbol{x}_i', \boldsymbol{x}_j)\right| \\ &\leq \sup_{\theta \in \mathscr{B}^p} \sum_{j \neq k} \Big(\left|\eta_\theta(\boldsymbol{x}_i, \boldsymbol{x}_j)\right| + \left|\eta_\theta(\boldsymbol{x}_i', \boldsymbol{x}_j)\right| \Big) \end{aligned} \tag{22}$$

Here, we assume that $w_\theta(\boldsymbol{x}) \leq s/N$ for any $\boldsymbol{x} \in \mathscr{X}$. We can enforce this constraint by thresholding the output of the approximating function $w_\theta(\cdot)$. Now the key question turns to investigating the upper bound for $\eta_\theta(\boldsymbol{x}_i, \boldsymbol{x}_j)$ and $\eta_\theta(\boldsymbol{x}_i', \boldsymbol{x}_j)$:

$$\eta_\theta(\boldsymbol{x}_i, \boldsymbol{x}_j) = w_\theta(\boldsymbol{x}_i) w_\theta(\boldsymbol{x}_j) \mathscr{K}(\boldsymbol{x}_i, \boldsymbol{x}_j) \leq \frac{s^2}{N^2} \sup_{\boldsymbol{x}, \boldsymbol{x}'} \mathscr{K}(\boldsymbol{x}, \boldsymbol{x}') = \frac{s^2 K}{N^2} \tag{23}$$

Based on this and the results from Equation 23, we can have the following:

$$\left|\Lambda(X) - \Lambda(X')\right| \leq \frac{2s^2 K}{N} \tag{24}$$

Applying the McDiamard inequality in Lemma 3, we can have the follows:

$$\Pr\Big(\left|\Lambda(X) - \mathbb{E}(\Lambda(X)\right| > \Delta\Big) \leq 2\exp\left(\frac{-N\Delta^2}{2s^4K^2}\right) = \frac{\delta}{(1 + 2/\varepsilon)^p} \tag{25}$$

From Equation 25, we can know the follows toward Δ:

$$\Delta = \frac{s^2 K}{\sqrt{N}} \sqrt{2\log(2/\delta) + 2p\log\left(1 + \frac{2}{\varepsilon}\right)} \tag{26}$$

Let Θ be an ε-cover of $\mathbb{B}^p$. This means that $\forall\theta \in \mathbb{B}^p \; \exists \; \theta' \in \Theta$ such that $\|\theta - \theta'\| \leq \varepsilon$. By Lemma 5.2 of[14] we have $|\Theta| \leq (1+2/\varepsilon)^p$. Using the above and union bounding over all elements of $\mathscr{A}$ and all k, we have:

$$\Pr\Big\{\forall\theta \in \Theta : \big|\Lambda(X) - \mathbb{E}(\Lambda(X))\big| \leq \Delta\Big\} \geq 1-\delta \tag{27}$$

Combining Equation 26 and 27, with at least probability of $1-\delta$ and for any $\theta \in \Theta$:

$$\mathscr{E}(\theta) - \mathscr{E}_X(\theta) \leq \mathbb{E}\Big\{\sup_{\theta\in\mathscr{B}^p}\big(\mathscr{E}(\theta) - \mathscr{E}_X(\theta)\big)\Big\} + \frac{s^2K}{\sqrt{N}}\sqrt{2\log(2/\delta) + 2p\log\Big(1+\frac{2}{\varepsilon}\Big)} \tag{28}$$

Next, we can investigate the upper bound for the $\mathbb{E}\Big\{\sup_{\theta\in\mathscr{B}^p}\big(\mathscr{E}(\theta) - \mathscr{E}_X(\theta)\big)\Big\}$. Through the symmetrization technical in[15], we can have the following:

$$\begin{aligned}\mathbb{E}\Big\{\sup_{\theta\in\mathscr{B}^p}\big(\mathscr{E}(\theta) - \mathscr{E}_{\boldsymbol{x}}(\theta)\big)\Big\} &\leq \mathbb{E}_{\boldsymbol{x},\sigma}\frac{1}{\lfloor N/2\rfloor}\sup_{\theta\in\mathscr{B}^p}\Big|\sum_{i=1}^{\lfloor N/2\rfloor}\sigma_i\eta_\theta\Big(\bar{\boldsymbol{x}}_i, \bar{\boldsymbol{x}}_{\lfloor\frac{N}{2}\rfloor+i}\Big)\Big| \\ &\leq \mathbb{E}_{\boldsymbol{x},\sigma}\frac{1}{\lfloor N/2\rfloor}\sup_{\theta\in\mathscr{B}^p}\Big|\sum_{i=1}^{\lfloor N/2\rfloor}\sigma_i\xi_i\Big| = R_N\end{aligned} \tag{29}$$

Here, we define $\sigma_1,\ldots\sigma_N$ to be a sequence of Rademacher variables and for each σ_i. we have $\Pr\{\sigma_i = 1\} = \Pr\{\sigma_i = -1\} = 0.5$. We further define $\xi_i = \mathscr{K}(\bar{\boldsymbol{x}}_i, \bar{\boldsymbol{x}}_{\lfloor N/2\rfloor+i})$ and $\bar{\boldsymbol{x}}_1,\ldots,\bar{\boldsymbol{x}}_N$ represents an independent and identically distributed copy of $\boldsymbol{x}_1,\ldots,\boldsymbol{x}_N$. The notation $\lfloor N/2\rfloor$ denotes the largest integer less than N and the notation R_N defines the empirical Rademacher complexity.

With this, we can have the following bound with at least a probability of $1-\delta$:

$$\mathscr{E}(\theta) - \mathscr{E}_X(\theta) \leq R_N + \frac{s^2K}{\sqrt{N}}\sqrt{2\log(2/\delta) + 2p\log\Big(1+\frac{2}{\varepsilon}\Big)} \tag{30}$$

Combining the bound in Equation 21, we can have with at least probability of $1-\delta$:

$$\mathscr{E}(\theta^*) - \mathscr{E}_X(\theta_{\text{opt}}) \leq R_N + \frac{s^2K}{\sqrt{N}}\sqrt{2\log(2/\delta) + 2p\log\Big(1+\frac{2}{\varepsilon}\Big)} \tag{31}$$

This completes the proof.

Theorem 3*:* Assumes the kernel function satisfying $\mathscr{K}(\boldsymbol{z},\boldsymbol{z}') \leq \mathscr{K}(\boldsymbol{z},\boldsymbol{z}) = K$ where $\boldsymbol{z} \neq \boldsymbol{z}'$. Define δ, c, c', λ as some constants

and $\delta \in (0,1)$, $\mathscr{N}_\infty(\lambda)$ as some constant depending on λ. Then with at least a probability of $1-\delta$, the following bound holds:

$$||\widehat{\mu}_M - \mu||_{\mathscr{H}} \leq \frac{2K\sqrt{2\log(6/\delta)}}{\sqrt{N}} + c'K\frac{\sqrt{2\log(6/\delta)}}{\sqrt{M}} + \sqrt{3\lambda} \times \left(\frac{4\sqrt{\mathscr{N}_\infty(\lambda)}\log(12/\delta)}{M} + \sqrt{\frac{2c v_{\mathbf{z}}\log(12/\delta)}{M}} \right) \tag{32}$$

where $v_{\mathbf{z}}^2 = \mathbb{E}(||\phi(\mathbf{z}_j) - \mathbb{E}(\phi(\mathbf{z}_j)||_{\mathscr{H}}^2)$ is established on the selected points.

Proof: Here, the empirical density estimation can be constructed as $\widehat{\mu}_N = \frac{1}{N}\sum_{i=1}^N \phi(\mathbf{x}_i)$ and $\mu = \int \phi(\mathbf{x})\, \mathrm{d}\mathbb{P}(\mathbf{x})$. We define the constructed kernel mean embedding based on the ground truth weights $\lambda_1^*, ...\lambda_M^*$ is as $\widehat{\mu}_{M^*} = \sum_{j=1}^M \lambda_j^* \phi(\mathbf{z}_j)$. To theoretically analyze the asymptotic behavior of $\widehat{\mu}_M$ under the proposed KTCS algorithm, we need to investigate an upper bound for the error term $||\widehat{\mu}_M - \mu||_{\mathscr{H}}$, which measures the deviation from μ, the kernel mean embedding at the population-level.

In order to generate an upper bound for $||\widehat{\mu}_M - \mu||_{\mathscr{H}}$, we can constitute the error decomposition as follows:

$$||\widehat{\mu}_M - \mu||_{\mathscr{H}} \leq ||\widehat{\mu}_{M^*} - \widehat{\mu}_M||_{\mathscr{H}} + ||\widehat{\mu}_{M^*} - \widehat{\mu}_N||_{\mathscr{H}} + ||\widehat{\mu}_N - \mu||_{\mathscr{H}} \tag{33}$$

First, let's look at the third component $||\widehat{\mu}_N - \mu||_{\mathscr{H}}$, which investigates the concentration ability of $\widehat{\mu}_N$ around its mean μ on a Hilbert space:

$$||\widehat{\mu}_N - \mu||_{\mathscr{H}} = \left\| \frac{1}{N}\sum_{i=1}^N \left[\phi(\mathbf{x}_i) - \mu\right] \right\|_{\mathscr{H}} \tag{34}$$

Furthermore, we can have $||\phi(\mathbf{x}_i) - \mu|| \leq 2\sup_{\mathbf{x}\in\mathscr{X}} ||\phi(\mathbf{x})|| = 2\sup_{\mathbf{x}\in\mathscr{X}} ||\mathscr{K}(\mathbf{x},\cdot)|| = 2K$, indicating that each individual term is bounded by $2K$. With this bounded term, we apply Lemma 1 with at least $1-\delta/3$ probability to have the following bound

$$||\widehat{\mu}_N - \mu||_{\mathscr{H}} \leq \frac{2K\sqrt{2\log(6/\delta)}}{\sqrt{N}} \tag{35}$$

Next, we move to investigate the bound toward the second term. Considering the underlying covariance structure among the selected points $\mathbf{z}_1, ..., \mathbf{z}_M$, we define $C := \int \phi(\mathbf{x}) \otimes_{\mathscr{H}} \phi(\mathbf{z}) d\mathbb{P}(\mathbf{z})$. To avoid singularity in C and C^{-1}, we introduce $C_\lambda = C + \lambda I$ where I is an identity matrix. And for the first component, we can define the following:

$$\begin{aligned} \forall \mathbf{z} \in \mathscr{Z}, \mathscr{N}_{\mathbf{z}}(\lambda) &:= \langle \phi(\mathbf{z}), C_\lambda^{-1}\phi(\mathbf{z}) \rangle_{\mathscr{H}} \\ \mathscr{N}_\infty(\lambda) &:= \sup_{\mathbf{z}\in\mathscr{Z}} \mathscr{N}_{\mathbf{z}}(\lambda) \end{aligned} \tag{36}$$

Based on these, we extend the second part as the follows:

$$\|\widehat{\mu}_N - \widehat{\mu}_{M^*}\|_{\mathscr{H}} = \|(I-P_M)\widehat{\mu}_N\|_{\mathscr{H}} = \|(I-P_M)(\widehat{\mu}_N - \widetilde{\mu}_M)\|_{\mathscr{H}} \tag{37}$$

where here $\widetilde{\mu}_M = \frac{1}{M}\sum_{j=1}^{M}\phi(\boldsymbol{z}_j)$ is defined to be the average of feature map aggregations.

Based on this C_λ matrix, we can generate the following bound:

$$\|(I-P_M)(\widehat{\mu}_N - \widetilde{\mu}_M)\|_{\mathscr{H}} \leq \left\|(I-P_M)C_\lambda^{1/2}\right\|_{\mathscr{H}} \left\|C_\lambda^{-1/2}\left(\widehat{\mu}_N - \widetilde{\mu}_M\right)\right\|_{\mathscr{H}} \tag{38}$$

In the proceeding part, we will derive an upper bound for the $\left\|C_\lambda^{-1/2}\left(\widehat{\mu}_N - \widetilde{\mu}_M\right)\right\|_{\mathscr{H}}$ part. As the selected points $\boldsymbol{z}_1,\ldots,\boldsymbol{z}_M$ are independent from the original dataset $\boldsymbol{x}_1,...,\boldsymbol{x}_N$. For the random variable $\boldsymbol{z}$, its expectation is calculated as follows:

$$\mathbb{E}\left[\phi(\boldsymbol{z})\right] = \int \phi(\boldsymbol{z})d\mathbb{P}(\boldsymbol{z}) = \frac{1}{N}\sum_{i=1}^{N}\phi(\boldsymbol{x}_i) = \widehat{\mu}_N \tag{39}$$

Based on the calculation of the $\mathbb{E}\left[\phi(\boldsymbol{z})\right]$, we can decompose the $\left\|C_\lambda^{-1/2}\left(\widehat{\mu}_N - \widetilde{\mu}_M\right)\right\|_{\mathscr{H}}$ as:

$$\left\|C_\lambda^{-1/2}\left(\widehat{\mu}_N - \widetilde{\mu}_M\right)\right\|_{\mathscr{H}} = \left\|\frac{1}{M}\sum_{j=1}^{M}\left(C_\lambda^{-1/2}\phi(\boldsymbol{z}_j) - C_\lambda^{-1/2}\widehat{\mu}_N\right)\right\|_{\mathscr{H}} \tag{40}$$

For simplicity, we define $\xi_j = C_\lambda^{-1/2}\phi(\boldsymbol{z}_j)$, and it is easy to verify that $\mathrm{E}(\xi_j) = C_\lambda^{-1/2}\widehat{\mu}_N$ for any $j = [1,...,M]$. With the application of the Cauthy-Schwartz inequality, we can derive the upper bound for the second order moment condition for random variable ξ_i

$$\begin{aligned}\mathbb{E}\left\|\zeta_i - \mathbb{E}\left[\zeta_i\right]\right\|_{\mathscr{H}}^2 &= \mathbb{E}\left\|C_\lambda^{-1/2}(\phi(\boldsymbol{z}_j) - \widehat{\mu}_N)\right\|_{\mathscr{H}}^2 \\ &\leq \mathbb{E}(\|C_\lambda^{-1/2}\|_{\mathscr{H}})\mathbb{E}(\|\phi(\boldsymbol{z}_j) - \widehat{\mu}_N\|_{\mathscr{H}}) = \nu_{\boldsymbol{z}}\,\mathrm{tr}(C_\lambda^{-1/2})\end{aligned} \tag{41}$$

Here, $\nu_{\boldsymbol{z}}^2 = \mathbb{E}(\|\phi(\boldsymbol{z}_j) - \widehat{\mu}_N\|_{\mathscr{H}}^2)$ is the variance of $\boldsymbol{z}$ in the RKHS as introduced in[16]. For simplicity, we define $c = \mathrm{tr}(C_\lambda^{-1/2})$. Then:

$$\mathbb{E}\left\|\zeta_i - \mathbb{E}\left[\zeta_i\right]\right\|_{\mathscr{H}}^2 \leq c\nu_{\boldsymbol{z}} \tag{42}$$

After considering the second moment condition, we investigate the upper bound for higher order moments $\mathbb{E}\left\|\zeta_i - \mathbb{E}\left[\zeta_i\right]\right\|_{\mathscr{H}}^p$,

when $p \geq 3$:

$$\begin{aligned}\mathbb{E}\,\|\zeta_i - \mathbb{E}\,[\zeta_i]\|_{\mathscr{H}}^p &= \mathbb{E}\,\|\zeta_i - \mathbb{E}\,[\zeta_i]\|_{\mathscr{H}}^2 \times \mathbb{E}\,\|\zeta_i - \mathbb{E}\,[\zeta_i]\|_{\mathscr{H}}^{p-2} \\ &\leq \nu\,\mathrm{tr}(C_\lambda^{-1/2})\,(\mathrm{ess\,sup}\,\|\zeta_i - \mathbb{E}\,[\zeta_i]\|_{\mathscr{H}})^{p-2} \\ &\leq \frac{1}{2}p!c\nu_z\left(2N_{Q,\infty}(\lambda)^{1/2}\right)^{p-2}\end{aligned} \tag{43}$$

as $\frac{1}{2}p! \geq 1$ always holds whenever $p \geq 3$. Combining the equations 42 and 43, we have:

$$\mathbb{E}\,\|\zeta_i - \mathbb{E}\,[\zeta_i]\|_{\mathscr{H}}^p \leq \frac{1}{2}p!c\nu_z\left(2N_{Q,\infty}(\lambda)^{1/2}\right)^{p-2} \tag{44}$$

holds for all $p \geq 2$. Applying Lemma 2, with at least a probability of $1-\delta/6$, we get the following upper bound:

$$\left\|C_\lambda^{-1/2}\,(\widetilde{\mu}_M - \widehat{\mu}_N)\right\|_{\mathscr{H}} \leq \frac{4\sqrt{\mathscr{N}_\infty(\lambda)}\log(12/\delta)}{M} + \sqrt{\frac{2c\nu_z\log(12/\delta)}{M}} \tag{45}$$

Adopting the results from[11], with at least probability of $1-\delta/6$:

$$\left\|(I-P_M)C_\lambda^{1/2}\right\|_{\mathscr{H}} \leq \sqrt{3\lambda} \tag{46}$$

Combining equation 45 and 46, with at least probability of $1-\delta/3$, the follow holds:

$$\|\widehat{\mu}_N - \widehat{\mu}_{M^*}\|_{\mathscr{H}} \leq \sqrt{3\lambda} \times \left(\frac{4\sqrt{\mathscr{N}_\infty(\lambda)}\log(12/\delta)}{M} + \sqrt{\frac{2c\nu_z\log(12/\delta)}{M}}\right) \tag{47}$$

In the last step, we investigate the upper bound for the first part. We define the parameters are trying to optimize the $\boldsymbol{\lambda}^* = \mathrm{argmin}_{\mathbf{w}}\,\|\Phi_M\mathbf{w} - \widehat{\mu}_N\|_{\mathscr{H}}$, this is equivalent with minimizing the associated MMD values. And $\boldsymbol{\lambda}^* = (\lambda_1^*, \ldots, \lambda_M^*)$. We can achieve this $\boldsymbol{\lambda}^* = (\Phi_M^*\Phi_M)^+\Phi_M^*\widehat{\mu}_N$. We work on the bound:

$$\|\widehat{\mu}_M - \widehat{\mu}_{M^*}\|_{\mathscr{H}} = \|\Phi_M\boldsymbol{\lambda} - \Phi_M\boldsymbol{\lambda}^*\|_{\mathscr{H}} = \left\|\sum_{j=1}^{M}(\lambda_j - \lambda_j^*)\phi(z_j)\right\|_{\mathscr{H}} \tag{48}$$

We make the assumption that $|\lambda_j - \lambda_j^*| \leq c'/M$. The rationale behind this assumption is when $\|\boldsymbol{\lambda}\|_1 = 1$ and there are M sub-components within the vector $\boldsymbol{\lambda}$, we aim to limit the deviation of λ_j from λ_j^* to prevent excessively large disparities. Large deviations $|\lambda_j - \lambda_j^*|$ in could potentially lead to trivial solutions, where a few points dominate all the weights within $\boldsymbol{\lambda}$. With

this, we have:

$$\| \sum_{j=1}^{M} (\lambda_j - \lambda_j^*) \phi(z_j) \|_{\mathscr{H}} \leq \| \frac{1}{M} \sum_{j=1}^{M} c' \phi(z_j) \|_{\mathscr{H}} \tag{49}$$

Meanwhile, according to the definitions of the feature map, the upper bound for the $\phi(z_j)$ is as $\phi(z_j) = \mathscr{K}(z_j, \cdot) \leq K$. Based on the Lemma 1, with at least a probability of $1 - \delta/3$:

$$\left\| \frac{1}{M} \sum_{j=1}^{M} c' \phi(z_j) \right\|_{\mathscr{H}} \leq c' K \frac{\sqrt{2 \log(2/\delta)}}{\sqrt{M}} \tag{50}$$

Based on equation 49, this implies with at least a probability of $1 - \delta/3$:

$$\| \widehat{\mu}_M - \widehat{\mu}_{M^*} \|_{\mathscr{H}} \leq c' K \frac{\sqrt{2 \log(6/\delta)}}{\sqrt{M}} \tag{51}$$

As we can have $\left(1 - \frac{\delta}{3}\right)^3 \geq (1 - \delta)$ so combining all the previous results in equation 35 equation 47 and equation 51, we can have the follows with at least probability of $1 - \delta$:

$$\| \widehat{\mu}_M - \mu \|_{\mathscr{H}} \leq \frac{2K \sqrt{2 \log(6/\delta)}}{\sqrt{N}} + c' K \frac{\sqrt{2 \log(6/\delta)}}{\sqrt{M}} + \sqrt{3\lambda} \times \left(\frac{4 \sqrt{\mathscr{N}_\infty(\lambda)} \log(12/\delta)}{M} + \sqrt{\frac{2 c \nu_z \log(12/\delta)}{M}} \right) \tag{52}$$

To examine the order of M, we introduce several additional assumptions about the parameters defined above. In particular, given the fact that:

$$\mathscr{N}_\infty(\lambda) = \sup_{z \in \mathscr{Z}} \mathscr{N}_z(\lambda) = \sup_{z \in \mathscr{Z}} \left\langle \phi(z), C_\lambda^{-1} \phi(z) \right\rangle \leq \frac{K^2}{\lambda} \tag{53}$$

So as for the third part of the equation 52, we obtain the bound as the follows:

$$\sqrt{3\lambda} \times \frac{4 \sqrt{\mathscr{N}_\infty(\lambda)} \log(12/\delta)}{M} \leq \frac{4 \sqrt{3} K \log(12/\delta)}{M} \tag{54}$$

Next, define $c = \mathrm{tr}(C_\lambda^{-1/2})$. Because the parameter c is a constant depends only on λ, we assume it obeys a polynomial decay of the form toward λ as $c = \mathrm{tr}(C_\lambda^{-1/2}) = c_\lambda \lambda^{1-\gamma}$, where c_λ is some constant and $\gamma \in (0, 1)$. Similar assumptions have been adopted in[11]. To relate λ to the size M we let λ inversely proportion to the size M. The larger the size M is, the smaller λ will

be. We assume $\lambda = (\frac{1}{M})^{1-\gamma}$, and with all of these, we can have the bound:

$$\sqrt{3\lambda} \times \sqrt{\frac{2c\nu_z \log(12/\delta)}{M}} = \frac{\sqrt{6c_\gamma}\log(12/\delta)}{M} \tag{55}$$

Combining the equation 54 and equation 55, we can have the following:

$$\sqrt{3\lambda} \times \left(\frac{4\sqrt{\mathcal{N}_\infty(\lambda)}\log(12/\delta)}{M} + \sqrt{\frac{2c\nu_z \log(12/\delta)}{M}} \right) = \mathcal{O}\left(\frac{1}{M}\right) \tag{56}$$

Furthermore, we assume the deviance between $\boldsymbol{\lambda}$ and $\boldsymbol{\lambda}^*$ satisfying $M|\lambda_j - \lambda_j^*| \leq \frac{1}{M^{1/2}}$, indicating that $c' = \mathcal{O}(M^{-1/2})$ Similar conditions and theoretical results can be found in[17]. Then the following will satisfy:

$$c'K\frac{\sqrt{2\log(6/\delta)}}{\sqrt{M}} = \mathcal{O}\left(\frac{1}{M}\right) \tag{57}$$

If we set the the size for subselected samples $M = \mathcal{O}(N^{1/2})$, then $\|\widehat{\mu}_M - \mu\|_{\mathcal{H}} = \mathcal{O}(\frac{1}{\sqrt{N}})$

Scaling Risk Confidence Interval

SR's corresponding confidence interval to better quantify the uncertainty of SR. Specifically, the 95% confidence interval for the log value of the scaling risk can be constructed as the follows:

$$\log SR \pm 1.96 \times \sqrt{\frac{\sum_{j=1}^M \lambda_j^2 r_j (1-r_j)}{(\sum_{j=1}^M \lambda_j r_j)^2}} \tag{58}$$

where $r_j = (I_j + 0.5)/2$. If the lower bound of the established confidence interval larger than 1, then we have 95% confidence to say the SUT could be riskier than human drivers.

Proof: The variance of the weighted failure count is constructed as the follows:

$$\mathrm{Var}\left[\sum_{j=1}^M \lambda_j I_j\right] = \sum_{j=1}^M \lambda_j^2 p_j (1-p_j) \tag{59}$$

Applying the delta method to $[\log \sum_{j=1}^M \lambda_j I_j]$ can have the follows:

$$\mathrm{Var}\left[\log \sum_{j=1}^M \lambda_j I_j\right] = \frac{\mathrm{Var}\left[\sum_{j=1}^M \lambda_j I_j\right]}{(\sum_{j=1}^M \lambda_j p_j)^2} = \frac{\sum_{j=1}^M \lambda_j^2 p_j (1-p_j)}{(\sum_{j=1}^M \lambda_j p_j)^2} \tag{60}$$

To avoid zero counts, we substitute $p_j = \frac{I_j + 0.5}{2}$ using the Haldane–Anscombe correction method[18]. So as the 95% confidence interval for the log value of the scaling risk can be constructed as the follows:

$$\log SR \pm 1.96 \times \sqrt{\frac{\sum_{j=1}^{M} \lambda_j^2 p_j (1 - p_j)}{(\sum_{j=1}^{M} \lambda_j p_j)^2}} \tag{61}$$

If the lower bound of the established confidence interval is larger than 1, then we have 95% confidence to say that the SUT could be riskier than human drivers. Similarly, if the upper bound smaller than 1, then the say the SUT could be safer than human drivers.

References

1. Li, T. & Yuan, M. On the optimality of gaussian kernel based nonparametric tests against smooth alternatives. *J. Mach. Learn. Res.* **25**, 1–62 (2024).
2. Mak, S. & Joseph, V. R. Support points. *The Annals Stat.* **46**, 2562–2592 (2018).
3. Zhang, J. *et al.* An optimal transport approach for selecting a representative subsample with application in efficient kernel density estimation. *J. Comput. Graph. Stat.* **32**, 329–339 (2023).
4. Kim, B., Khanna, R. & Koyejo, O. O. Examples are not enough, learn to criticize. criticism for interpretability. *Adv. Neural Inf. Process. Syst.* **29** (2016).
5. Joseph, V. R., Dasgupta, T., Tuo, R. & Wu, C. J. Sequential exploration of complex surfaces using minimum energy designs. *Technometrics* **57**, 64–74 (2015).
6. Chen, Y., Gao, Q. & Wang, X. Inferential wasserstein generative adversarial networks. *J. Royal Stat. Soc. Ser. B: Stat. Methodol.* **84**, 83–113 (2022).
7. Guo, F. Statistical methods for naturalistic driving studies. *Annu. Rev. Stat. its Appl.* **6**, 309–328 (2019).
8. Feng, S. *et al.* Dense reinforcement learning for safety validation of autonomous vehicles. *Nature* **615**, 620–627 (2023).
9. Pinelis, I. Optimum bounds for the distributions of martingales in banach spaces. *The Annals Probab.* 1679–1706 (1994).
10. Yurinsky, V. *Sums and Gaussian vectors* (Springer, 2006).
11. Chatalic, A., Schreuder, N., Rosasco, L. & Rudi, A. Nyström kernel mean embeddings. In *International Conference on Machine Learning*, 3006–3024 (PMLR, 2022).

12. Rudi, A., Camoriano, R. & Rosasco, L. Less is more: Nyström computational regularization. *Adv. Neural Inf. Process. Syst.* **28** (2015).

13. Rosén, B. On inclusion probabilities for order πps sampling. *J. Stat. Plan. Inference* **90**, 117–143 (2000).

14. Eldar, Y. C. & Kutyniok, G. *Compressed sensing : theory and applications* (Cambridge University Press, Cambridge, United Kingdom, 2012).

15. Bartlett, P. L. & Mendelson, S. Rademacher and gaussian complexities: Risk bounds and structural results. *J. Mach. Learn. Res.* **3**, 463–482 (2002).

16. Wolfer, G. & Alquier, P. Variance-aware estimation of kernel mean embedding. *J. Mach. Learn. Res.* **26**, 1–48 (2025).

17. Liu, W., Yu, X., Zhong, W. & Li, R. Projection test for mean vector in high dimensions. *J. Am. Stat. Assoc.* 1–13 (2022).

18. Lachin, J. M. *Biostatistical methods: the assessment of relative risks* (John Wiley & Sons, 2014).
